# Comprehensive Eco-Environment Quality Index Model with Spatiotemporal Characteristics

**DOI:** 10.3390/s22249635

**Published:** 2022-12-08

**Authors:** Ning Li, Jiayao Wang

**Affiliations:** 1 College of Geography and Environmental Science, Henan University, Zhengzhou 450046, China; 2 Henan Industrial Technology Academy of Spatio-Temporal Big Data, Henan University, Zhengzhou 450046, China; 3 Laboratory of Geospatial Technology for the Middle and Lower Yellow River Regions, Ministry of Education, Henan University, Kaifeng 475004, China; 4 Henan Technology Innovation Center of Spatio-Temporal Big Data, Henan University, Zhengzhou 450046, China

**Keywords:** ecological environment quality, temporal accuracy, spatial accuracy

## Abstract

Ecological environment assessment, which forms the basis for the survival and development of human society, is a crucial measure for the sustainable development of society and economy. However, current quantitative assessment models such as *EI*, *EQI*, *RSEI* et al. are insufficient to meet the requirements of dynamic research in large areas, long time series and dense time intervals. Therefore, in this paper, we constructed the comprehensive ecological environment quality index model *S_CEQI_* that can meet these needs by applying the remote sensing big data as the data source. The *S_CEQI_* aggregated the ecological indices *NDVI*, *NDBSI*, *Lst* and *Wet* by full-sequence dynamic dimensionless, automated principal component analysis and multi-temporal average method. In order to verify the spatial and temporal accuracy of the model, we took Henan Province as an example to compare the result of *S_CEQI_* with that of *EI*, *EQI*, *RSEI* and proved that *S_CEQI_* performed better in both time and space. Also, we verified the superiority of time, space, and precision of *S_CEQI_* with profiles, samples, and cluster analysis.

## 1. Introduction

According to the 6th IPCC Assessment Report, the global average temperature would increase 1.5 degrees Celsius by the early 2030s under the current emission scenario [1], which would have important impacts on ecological environment. The influence of human activities also results in complicated and multi-dimensional changes of ecological environment [2]. The United Nations has proposed the Millennium Ecosystem Assessment Plan [3], 2030 Sustainable Development Goals [4], and the initiative Decade of Ecosystem Restoration [5] aiming at a multi-level and comprehensive assessment of the global ecosystem. Therefore, it is necessary to integrate ecological big data of multi-source and multi-scale to carry the quantitative research of time-intensive and spatial-refined on the comprehensive ecological environmental quality assessment. The development of remote sensing and cloud computing technologies provides the possibility for the evaluation. How to build an ecological environment quality evaluation model suitable for remote sensing big data to carry out comprehensive ecological environment research in large areas, long time series, and intensive time intervals is the focus of this paper.

At present, the quantitative ecological environment quality measurement models are (1) a single proxy index such as vegetation cover change [6,7,8], heat island effect [9,10], landscape pattern index [11,12], ecological service value [13,14,15,16,17], ecological contribution rate [18], carbon emission [10], normalized vegetation index (*NDVI*) [19,20], enhanced vegetation index [21], and net primary productivity of vegetation [22], (2) ecological index (*EI*) model in The Technical Specification of Ecological Environmental Status Assessment (HJ 192–2015) [23] issued by the Ministry of Ecology and Environmental Protection of China, (3)the eco-environmental quality index (*EQI*) proposed by Li Xiaowen, which is based on high-resolution land use/land cover data and the corresponding weight [24] to measure the eco-environmental quality, (4) the remote sensing ecological environment index (*RSEI*) [25,26] proposed by Xu Hanqiu calculate the eco-environment index-based remote-sensing image, (5) the improved models of *RSEI* such as the improvement of *RSEI* for time series analysis [27], the improvement of *RSEI* for the instability [28], the RO-RSEI which is the improvement for the ecological indicators [29], the ARSEI which is the improvement for the ecological indicators [30].

Although the current models for quantitatively measuring the regional ecological environmental quality can reflect the regional ecological environment status, they can hardly reflect the spatial heterogeneity of the ecological environment objectively. The proxy indices could only reflect one aspect of the ecological environment and are not enough to represent the comprehensive ecological environment. The *EI* and *EQI* models rely on LUCC data whose accuracy of the secondary land use classification is not high enough [31]. In addition, the weight corresponding to the land use type is subjective and fixed so that it could not distinguish the spatial differentiation of the ecological environment of the same land use type. What’s more, the recalculation time period of the two models is relatively long due to the limitation of the temporal resolution of the LUCC data source. The *RSEI* is the most popular and widely used remote sensing ecological environment model due to the easy access to indicators that can be obtained through the remote sensing images with medium to high resolution. However, it also has some problems such as (1) static non-depersonalization method leads to the research area of the model could not exceed the scene of the image, and the results of different-phase images could not be compared effectively, (2) the model is unstable result from the PCA index aggregation method who would obtain two opposite results randomly, (3) using *RSEI* result of a single image to represent the regional annual ecological environment status ignores the phenological characteristics of vegetation. Although the improved models of *RSEI* have optimized the model, so far, there is still no quantitative ecological environment measurement model that can use remote sensing big data to carry out research on large areas, long time series, and intensive time intervals.

With the deepening and perfection of research on eco-environment, the evaluation method of ecological environmental quality has shifted from the traditional simple and single estimation to spatiotemporal dynamic assessment. In this paper, we constructed a new comprehensive remote sensing ecological environment quality index model *S_CEQI_* with spatial and temporal characteristics by improving the data source, dimensionless method, index aggregation method of *RSEI* and adding the multi-temporal mean value method. Remote sensing big data makes the model possible to conduct intensive time interval research, the full sequence dimensionless method enables the model to carry large-scale and long-term time-series research, and automated index aggregation method solves the problem of model instability and enables the model to perform batch calculations automatically. The multi-temporal mean value method solves the contingency problem result from the result of single image. It is more convincing and credible to use the average value of the calculation results of multi-temporal images in one year to represent the ecological environment state in that year than a single image. Finally, taking Henan Province as an example, we compared the results of the *EI*, *EQI*, *RSEI*, and *S_CEQI_*, to verify the temporal and spatial superiority of *S_CEQI_* through profile analysis and sample point analysis. In terms of space, the model can well distinguish the spatial heterogeneity of the ecological environment index between different ecosystems and the same ecosystem; in terms of time, the model can express the ecological environment differences of plants in different phenological stages.

## 2. Materials and Methods

### 2.1. Study Area and Data Sources

#### 2.1.1. Study Area

**Figure 1:**
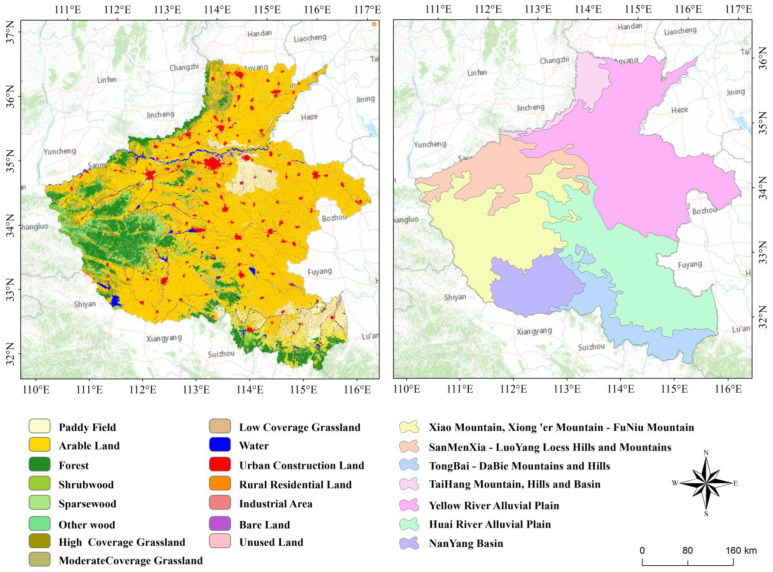
LUCC and geomorphological map of Henan Province.

Henan Province (31°23′–36°22′ N, 110°21′–116°39′ E) is located in the transition zone between the middle and lower reaches of the Yellow River, with a total area of ~167,000 km^2^. The landform types are mountains, hills, and plains from west to east. And the ecosystems are forest, grass, and cultivated land from west to east (Figure 1, left). The mountainous area of the Henan Province is ~44,000 km^2^, hilly area is ~29,600 km^2^, and plain area is ~93,000 km^2^, accounting for 26.59%, 17.72%, and 55.69% of the total area, respectively (Figure 1, right). The main LUCC types in the Henan Province are arable, forest, and construction lands, accounting for more than 90% of the total area. The arable accounts for 55.71% in 2019, which decreased by 10.43% between 1995 and 2019, mainly distributed in the Huang-Huai-Hai Plain and Nanyang Basin. The forest accounts for 24.08%, which increased by 5.26% between 1995 and 2019, mainly distributed in the mountainous and hilly areas in western Henan, the Taihang Mountainous and hilly area in northwestern Henan, and the Dabie-Tongbai Mountains in southern Henan. The construction land accounts for 15.74%, which increased by 6.04% between 1995 and 2019, mainly distributed in the Yellow River alluvial plain and the Huai River alluvial plain. The total population of Henan Province is 109.06 million with a 653 people/km^2^ population density in 2019, of which the urban population is 56.39 million, accounting for 51.71%, and the rural population is 52.67 million, accounting for 48.29%. The GDP increased from 298.837 billion yuan in 1995 to 4805.586 billion yuan in 2019, with an increase of 16.08 times in the past 25 years.

Henan Province is located in the transition zone between the subtropical zone and the warm temperate zone, and its climate has obvious transitional characteristics. It has complex biota and rich biodiversity. The complex landform types and climate change characteristics resulted in scarcity and uneven distribution of water resources, and frequent drought and flood disasters. Besides, rapid urbanization has disturbed the natural landscape and ecosystems of Henan Province. As a result, the ecological environment has large spatial differences and low carrying capacity in Henan Province [32].

#### 2.1.2. Data Sources

**Figure 2:**
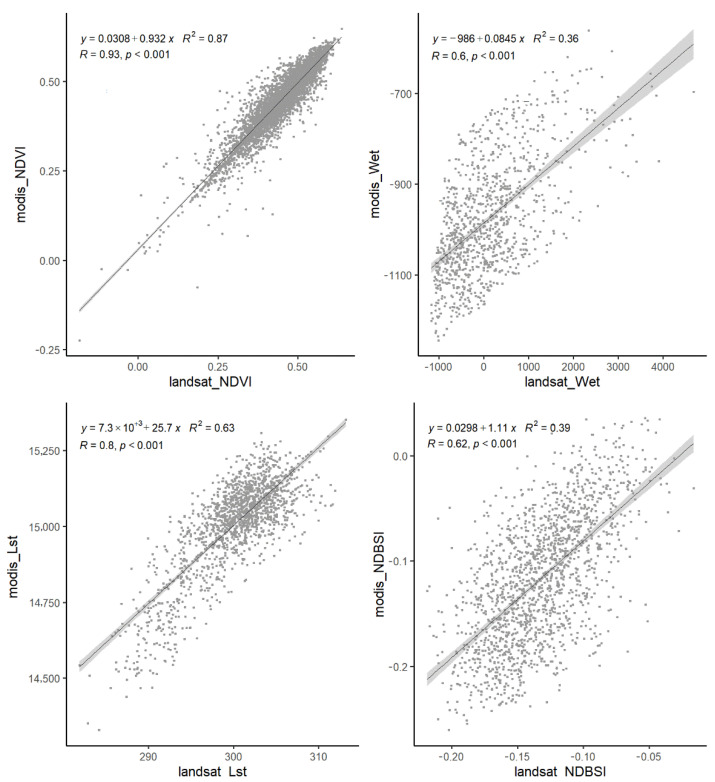
Precision verification of indicators (*NDVI*, *Wet*, *Lst*, *NDBSI*).

Landsat remote sensing image has been applied in many studies due to its long sequence and high spatial resolution. In this study, Landsat 5 and Landsat 8 images of Tier 1 from the United States Geological Survey (USGS) were used to calculate the indicators of *NDVI*, *NDBSI*, *Wet*, and *Lst* (Table 1). Additionally, the *Lst* indicator used water vapor data from the NCEP/NCAR Reanalysis Project [33], LUCC data used in *EI* and *EQI* model was from the Data Center of Middle&Lower Yellow River Regions, National Earth System Science Data Center, National Science&Technology Infrastructure of China (http://henu.geodata.cn, accessed on 17 March 2022), and its secondary classification accuracy is 91.19% [31]. We compared the mean value of *NDVI*, *Lst* of 2019 with the publicly available datasets (Figure 2), and the correlation coefficients are both greater than 0.8. Since there are no available public datasets of *NDBSI* and *Wet*, we calculated the two indices using Modis and Landsat data, respectively, and analyzed their correlation. Although the sensors and spatial resolutions of Modis and Landsat data are different, *NDBSI* and *Wet* still showed a significant correlation with a correlation coefficient greater than 0.6.

### 2.2. Methods

#### 2.2.1. EI Model

*EI* is the weighted summation of a series of indicators that reflect the regional ecological environmental situation such as biological abundance, vegetation cover, water density, land stress, and pollution load as well as the restrictive indicator of environmental limitation index. The weight of each index is shown in Table 2.

The *EI* model can be expressed as follows:

(1) $$\begin{array}{c} EI=0.35\times BA+0.25\times NDVI+\\ 0.15\times WD+0.15\times \left(100-LS\right)\\ +0.10\times \left(100-PL\right)+EL \end{array}$$

and biological abundance (*BA*) can be calculated using Equation (2)

(2) BA=(BI+HQ)/2

where HQ stands for habitat quality index and *BI* refers to biological diversity. Before updating the biodiversity index, biological abundance could be calculated from *HQ* as follows:

(3) $$\begin{array}{c} HQ=A_{bio}\times (0.35\times A_{forest}+0.21\times A_{grass}+0.28\times A_{water}\\ +0.11\times A_{farmland}+0.04\times A_{construction}+0.01\times A_{unused})/A \end{array}$$

where $A_{bio}$ is the normalized coefficient of habitat quality index; $A_{forest}$, $A_{grass}$, $A_{water},$ $A_{farmland}$, $A_{construction},$ and $A_{unused}$ denote the area of forest, grass, water, farmland, construction, and unused land, respectively; and *A* indicates the total study area.

Further, *NDVI* could be expressed as follows:

(4) $$NDVI=NDVI_{mean}=A_{veg}\times \frac{\sum_{i=1}^{n}P_{i}}{n}$$

where $A_{veg}$ is the normalized differential vegetation index, n is the number of pixels in the region, and $P_{i}$ is the mean value of the max *NDVI* from May to September.

*WD* was calculated as follows:

(5) $$WD=\frac{\frac{A_{riv}\times S_{riv}\times A_{lak}+A_{res}\times S_{res}}{S}}{3}$$

where $A_{\mathrm{riv}}$, $S_{\mathrm{riv}}$, and $A_{\mathrm{res}}$ are the river length, water area (lake, reservoir, river canal and offshore), and normalization coefficient of water resources, respectively; $S_{riv}$, $S_{\mathrm{lak}}$, and $S_{\mathrm{res}}$ are the river length, water area (lakes, reservoirs, river channels, and offshore waters), and water resources, respectively; and S is the study area.

*LS* can be calculated using Equation (6):

(6) $$LS=\frac{A_{reo}\times \left(0.4\times S_{ero1}+0.2\times S_{reo2}+0.2\times S_{reo3}+0.2\times S_{reo4}\right)}{S}$$

where $A_{\mathrm{reo}}$ is the normalized coefficient of land stress index and $S_{reo1}$, $S_{ero2}$, $S_{\mathrm{ero}3}$, and $S_{ero4}$ are the areas of severe soil erosion, moderate soil erosion, construction land, and other land stress indices, respectively.

*PL* can be calculated using Equation (7):

(7) $$\begin{array}{c} PL=\frac{0.2\times A_{COD}\times E_{COD}}{P}+\frac{0.2\times A_{NH3}\times E_{NH3}}{P}+\frac{0.2\times A_{SO2}\times E_{SO2}}{P}\\ +\frac{0.1\times A_{YHC}\times E_{YHC}}{P}+\frac{0.2\times A_{NOX}\times E_{NOX}}{P}+\frac{0.1\times A_{SOL}\times E_{SOL}}{P} \end{array}$$

where $A_{COD}$, $A_{NH3}$, $A_{SO2}$, $A_{YFC}$, $A_{NOX}$, and $A_{SOL}$ are the normalized coefficients of chemical oxygen demand, ammonia nitrogen, sulfur dioxide smoke (powder) dust, nitrogen oxide, and solid waste, respectively; $E_{COD}$, $E_{NH3}$, $E_{SO2}$, $E_{YFC}$, $E_{NOX}$, and $E_{SOL}$ refer to chemical oxygen demand, ammonia nitrogen, sulfur dioxide smoke (powder) dust, nitrogen oxide, and solid waste emissions, respectively; and P indicates annual precipitation in the study area.

Environmental restriction index refers to restrictive indicator of ecological environment conditions, which means that the types of ecological environment conditions are restricted and adjusted according to the ecological damage and environmental pollution, such as sudden environmental events, which seriously affect the production and living safety of human settlements in a region.

According to the eco-environmental status index, the eco-environmental status is divided into five levels, excellent, good, medium, inferior, and poor. The levels can be further divided as Table 3:

#### 2.2.2. EQI Model

The *EQI* [24] model is based on expert scores of the different land use/land cover secondary classification type (Table 4) to analyze the quantity and spatial characteristics of regional ecological environment. It can be expressed as follows:

(8) $$EQI_{t}=\sum_{i=1}^{n}LUA_{i,t}\times LUC_{i}/SA$$

where $EQI_{t}$ stands for regional environment quality during period *t*, $LUA_{i,t}$ represents the area of secondary land use type *i* in period *t*, $LUC_{i}$ refers to the eco-environmental quality index of the corresponding secondary land use type *I,* and SA indicates the total study area.

#### 2.2.3. RSEI Model

The *RSEI* model was proposed by Xu [25]. It uses remote sensing data to completely evaluate regional ecological environment instead of land use data. The *RSEI* model is expressed as Equations (9) and (10):

(9) $$RSEI_{0}=PC1\left[f\left(NDVI_{s},NDSI_{s},Wet_{s},Lst_{s}\right)\right]$$

(10) $$RSEI=\frac{RSEI_{0}-RSEI_{min}}{RSEI_{max}-RSEI_{min}}$$

Due to instability of *RSEI*, Equation (11) is often used instead of Equation (9):

(11) $$RSEI_{0}=1-PC1\left[f\left(NDVI_{s},NDSI_{s},Wet_{s},Lst_{s}\right)\right]$$

where $RSEI_{0}$ is the ecological environmental index before standardization; RSEI is the ecological environmental index after standardization; $NDVI_{s},NDSI_{s},Wet_{s},\;\mathrm{and}\;Lst_{s}$ are the four standardized indices in *RSEI* model; $PC1\left[f\left(NDVI_{s},NDSI_{s},Wet_{s},Lst_{s}\right)\right]$ indicates the first principal component; and $RSEI_{min}$ and $RSEI_{max}$ stand for the minimum and maximum values of $RSEI_{0}$, respectively. The four indicators can be calculated as follows:

1. NDVI

*NDVI* [25] reflects the condition of land cover vegetation. In this study, it represents the greenness indicator of ecological environmental quality. Greenness can be expressed as follows:

(12) $$NDVI=\frac{nir-red}{nir+red}$$

where *NDVI* represents the greenness indicator, *nir* refers to the near-infrared band of remote sensing images, and *red* stands for the red band.

2. NDBSI

*NDBSI* reflects the condition of the impervious surface, which is the average of the bare soil index and construction index. It represents the dryness indicator of the ecological environmental quality [25]. The formula for dryness is as follows:

(13) $$NDBSI=\frac{\left[\frac{\left(red+swir1\right)-\left(nir+green\right)}{\left(red+swir1\right)+\left(nir+green\right)}+\frac{2*\frac{nir}{nir+red}-\left(\frac{red}{red+green}+\frac{blue}{blue+nir}\right)}{2*\frac{nir}{nir+red}+\left(\frac{red}{red+green}+\frac{blue}{blue+nir}\right)}\right]}{2}$$

where *red* represents the *red*-light band in the visible light band of remote sensing image, *green* refers to the *green*-light band, *blue* represents the *blue*-light band, *swir* stands for the mid-infrared band, and *nir* indicates the near-infrared band.

3. Wet

The moisture component of tasseled cap transformation reflects the ground moisture condition, and it characterizes the soil moisture state. In this study, *Wet* represents the humidity indicator of ecological environmental quality [25], which can be expressed as follows based on the TM sensor:

(14) $$\begin{array}{c} \mathrm{Wet}=0.0315\times nir+0.2021\times red+0.3102\times blue\\ +0.1594\times green-0.6806\times swir1-0.6109\times swir2 \end{array}$$

The humidity indicator can be expressed as follows based on the OLI/TIRS sensor:

(15) $$\begin{array}{c} \mathrm{Wet}=0.1511\times nir+0.1973\times red+0.3283\times blue\\ +0.3407\times green-0.7117\times swir1-0.4559\times swir2 \end{array}$$

where *red* refers to *red*-light band, *green* represents the *green*-light band, *blue* stands for the *blue*-light band, *swir* indicates the mid-infrared band, and *nir* refers to the near-infrared band in the visible light band of remote sensing image.

4. Lst

The surface temperature *Lst* reflects the temperature of the ground. In this study, the surface temperature *Lst* represents the heat indicator of eco-environmental quality [25]. The single-channel algorithm was used to estimate surface temperature [34,35] as follows:

(16) $$Lst=\gamma \left[\frac{1}{\varepsilon_{i}}\left(\varphi_{1}L_{i}+\varphi_{2}\right)+\varphi_{3}\right]+\delta$$

(17) $$\gamma \approx \frac{T_{i}{}^{2}}{b_{r}L_{i}}$$

(18) $$\delta \approx T_{i}-\frac{T_{i}{}^{2}}{b_{r}}$$

where *Lst* represents the heat indicator; $\varepsilon_{i}$ refers to surface-specific emissivity; γ and δ are correlation coefficients of the Planck equation; $b_{r}$ = $\frac{c_{2}}{\lambda }$, $c_{2}$ = 1.4387685, λ indicates the effective wavelength of thermal infrared band; $L_{i}$ represents radiant brightness on the star; $T_{i}$ stands for brightness temperature on the star; $\varphi_{1}$, $\varphi_{2}$,$\;\mathrm{and}\;\varphi_{3}$ are three atmospheric function parameters; and w is the atmospheric water vapor content. The expression of *w* based on the OLI/TIRS sensor was as follows:

(19) $$\left[\begin{array}{c} \;\varphi_{1}\\ \varphi_{2}\\ \varphi_{3} \end{array}\right]=\left[\begin{array}{ccc} \begin{array}{c} 0.04019\\ -0.38333\\ 0.00918 \end{array} & \begin{array}{c} 0.02916\\ -1.50294\\ 1.36072 \end{array} & \begin{array}{c} 1.01523\\ 0.20324\\ -0.27514 \end{array} \end{array}\right]\left[\begin{array}{c} w^{2}\\ w\\ 1 \end{array}\right]$$

The expression of *w* based on the TM sensor was as follows:

(20) $$\left[\begin{array}{c} \varphi_{1}\\ \varphi_{2}\\ \varphi_{3} \end{array}\right]=\left[\begin{array}{ccc} \begin{array}{c} 0.06982\\ -0.51041\\ -0.05457 \end{array} & \begin{array}{c} -0.03366\\ -1.20026\\ 1.52631 \end{array} & \begin{array}{c} 1.04896\\ 0.06297\\ -0.32136 \end{array} \end{array}\right]\left[\begin{array}{c} w^{2}\\ w\\ 1 \end{array}\right]$$

#### 2.2.4. Analysis of Current Models

According to the theoretical basis of *EI* model and previous studies, it is known that the *EI* model can reflect the regional comprehensive ecological environment correctly. Although *EI* model has been widely used, it has some shortcomings: (1) It is difficult to obtain the data of chemical oxygen demand, ammonia nitrogen, sulfur dioxide, smoke (powder) dust, nitrogen oxide, solid waste and total nitrogen emissions, and pollution load index. (2) The *EI* model takes administrative unit as a statistical unit, which is not fine enough in space, and the difference in ecological environment quality in the same unit cannot be expressed. (3) The weight of indices in *EI* model is artificially determined, which is subjective and fixed without geographical distinction. (4) The biological abundance index in *EI* model accounts for 0.35 weight, and it is calculated depending on the secondary land use classification data. However, at present, the second-level classification accuracy is not high enough [31].

According to the theoretical basis of *EQI* models and previous studies, it is known that the principle of *EQI* model is to assign weight to the land parcel based on the corresponding secondary land use types. The *EQI* model can quantitatively evaluate regional comprehensive ecological environment quality, and it is widely used to calculate the eco-environmental effects of land use change. However, the *EQI* model has some limitations in application. (1) The expert assignment method and analytical hierarchy process are both subjective. (2) The model results totally depend on land use data, which require high accuracy of land use secondary classification as well as the *EI* model. (3) Assigning weight of land use types ignores differences in lands with the same type of LUCC and their geographical differences in space, for example, the difference in cultivated land ecological environmental quality between plains and mountains, the difference in ecological environment quality between deciduous and evergreen forests and between broadleaf and coniferous forests, and the difference in ecological environment quality between the construction land in mountains and plains.

The *RSEI* model can overcome the restrictions of administrative divisions and available data and evaluate regional comprehensive ecological environment quality quantitatively, objectively, and accurately. It is the most widely used model in academic circles; however, it has some limitations. (1) In previous studies, a single image of October with less clouds were used to measure the annual sensing ecological environment quality, resulting in random results. As a result, the yearly results could not be compared effectively. Additionally, a single image cannot represent the ecological status of the region throughout the year. For example, in October, the ecological environmental index of forest was the highest and that of cultivated land was the lowest. In fact, the ecological status of forest was only slightly higher than that of cultivated land most of the time, and the ecological status of winter wheat was better than that of deciduous forest in winter. Thus, a single image cannot reflect the phenological characteristics of plants and it cannot represent the ecological environmental state of the year or quarter; it can only reflect the current ecological environmental state. (2) The *RSEI* model is only suitable for small study areas. Studies on *RSEI* model to calculate remote sensing *EQI* are concentrated in small study areas such as cities, municipal districts, districts, counties, nature reserves, and other parks. This is because remote sensing images are instantaneous and the calculation results of different images at different times differ considerably, resulting in obvious “cliff” at the edge of two adjacent images. (3) *RSEI* can objectively evaluate the differences in ecological status in the same area at the same time, and the results obtained from images in the same area at different times are not comparable. This is because *RSEI* uses a static dimensionless method. (4) The *RSEI* model is unstable. In the application of *RSEI* model, two opposite models are used. Some scholars calculated the value of “1-*PC*1” as an ecological environmental index and some scholars directly use the standardized value of *PC*1 as the regional eco-environment index. Our previous study revealed that it is the direction of the eigenvector that causes this phenomenon.

#### 2.2.5. Improved Model S_CEQI_

**Figure 3:**
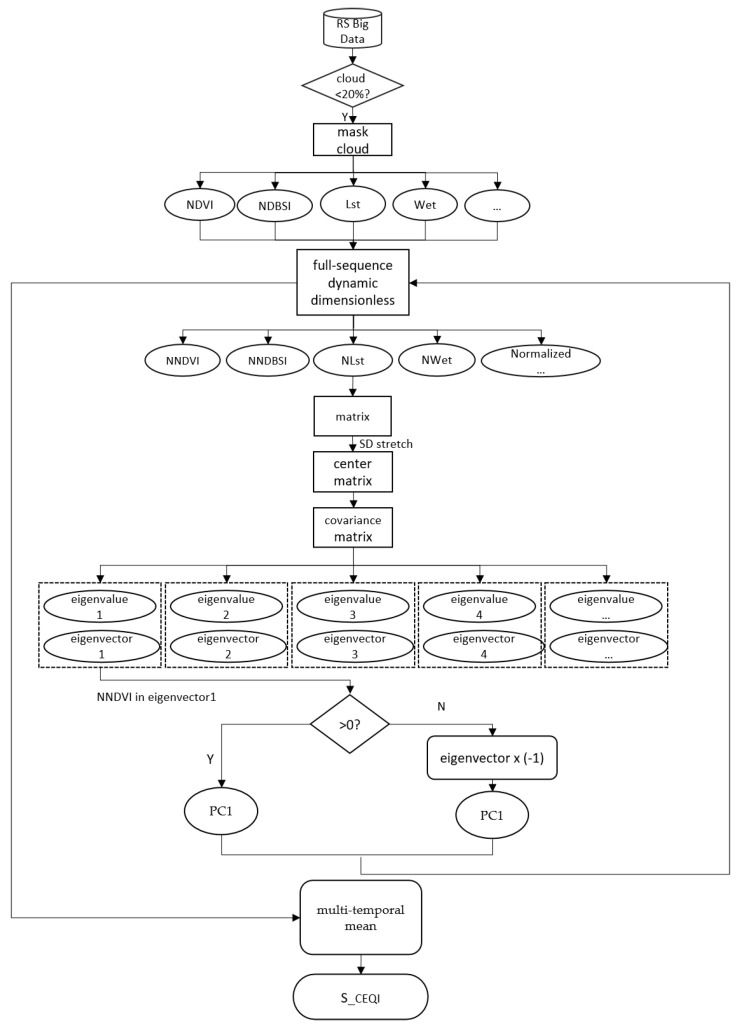
The flowchart of the *S_CEQI_*.

*RSEI* is a more precise, popular, and objective model than *EI* and *EQI*. However, because the model uses a single remote sensing image to calculate the eco-environmental quality, the result of instantaneous image lacks spatiotemporal characteristics. In this study, we improved the *RSEI* model using the remote sensing big data instead of a single image, replacing the static dimensionless method with a full sequence dimensionless method and improving the traditional human involvement aggregation method to automation indicator aggregation method. Based on these improvements, we renamed the remote sensing ecological environment index model with spatio-temporal characteristics as “*S_CEQI_*.” The flowchart of the model is shown in Figure 3.

##### Improvement of Data Source

Big data is important to support the achievement of Sustainable Development Goals (SDGs). High-quality and fine-grained data is critical to transform the SDGs into tools for making good decisions. Studies on SDGs are likely to be supported by big data, which spans multiple data sources and can fill data gaps in multiple ways, complementing the existing sustainability assessment tools and indicators. Current studies on ecological environment quality are relatively simple and not thorough owing to the lack of enough data sources. Cloud computing technology provides technical possibilities for using big data for ecological environmental quality assessment. As remote sensing big data has characteristics of long life and multi-scale, it can be used to conduct ecological environmental quality studies with long-time series, multi-scale, and dense time interval dynamically together with a cloud computing platform. The application of remote sensing big data uses a single image to solve the problem of measuring the overall ecological environmental quality in the traditional quantitative ecological environmental quality model and to dynamically measure the regional ecological environmental status in time and space.

As a data source, remote sensing big data requires automated batch processing capability of the measurement models. To meet this requirement, this study improved the index dimensionless and aggregation methods and added the multi-temporal mean method and real ecological environmental conditions.

##### Improvement of Dimensionless Method

The purpose of the indicator dimensionless method is to eliminate the differences in units and magnitudes of different indicators, and it is an important part of comprehensive evaluation. The reliability of the dimensionless method determines the credibility of comprehensive evaluation results. Comprehensive evaluation includes static comprehensive evaluation and dynamic comprehensive evaluation, and different evaluation methods use different dimensionless methods. The static dimensionless method causes distortion of evaluation results to a certain extent. Aiming at the problems of *RSEI* that the study area cannot be larger than the Landsat image area and the results at different times are not related or comparable, as a result of the static dimensionless method, we proposed a full-sequence dimensionless method to establish the relationship between images in different areas and periods. The full sequence method is a simple and intuitive index dimensionless method based on spatiotemporal data, which can be well-extended to various static dimensionless methods while retaining the original numerical characteristics of the method [36]. The full-sequence dimensionless method expression is as follows:

(21) $$x_{ij}^{*}\left(t_{k}\right)=c+\frac{x_{ij}\left(t_{k}\right)-\underset{i,k}{\min}\left\{x_{ij}\left(t_{k}\right)\right\}}{\underset{i,k}{\max}\left\{x_{ij}\left(t_{k}\right)\right\}-\underset{i,k}{\min}\left\{x_{ij}\left(t_{k}\right)\right\}}\times d,\;k=1,2,3,\cdots ,N$$

where $x_{ij}^{*}\left(t_{k}\right)$ represents the dimensionless result of jth at time $t_{k}$, $x_{ij}\left(t_{k}\right)$ represents the original data, $\underset{i,k}{\max}\left\{x_{ij}\left(t_{k}\right)\right\}$ and $\underset{i,k}{\min}\left\{x_{ij}\left(t_{k}\right)\right\}$ represent the maximum and minimum values, respectively, and c and d are constants, representing the translation of dimensionless result and scaling of dimensionless index, respectively, which can be determined according to the actual situation of the study.

##### Improvement of Indicator Aggregation Method

The weight of each indicator in *RSEI* is determined by *PC*1 of principal component analysis. And the eigenvector of *PC*1 is the weight of the indices. However, it can only determine the weight value but not its direction due to the non-uniqueness of the eigenvectors. So, in this paper, we improved the aggregation method according to the effect (improvement or deterioration) of ecological indicators on the ecological environment. The weights of indicators that have a positive effect on the ecological environment are positive, and the weights of indicators that have a negative impact on the ecological environment are negative. The improved indicator aggregation method is unique and can aggregate ecological indicators automatically and intelligently, which is the basis for big data to be used in ecological environment assessment. The aggregation method is as follows:

If

(22) $$X={\left[x_{1},\;x_{2},\;\ldots x_{m}\right]}^{T}$$

(23) $$A_{i}={\left[v_{i1\;}v_{i2\;\cdots \;}v_{im\;}\right]}^{T}$$

Then

(24) $$F_{i}=A_{i\;}^{T}X$$

where $x_{1},\;x_{2}$, …$x_{m}$ are ecological indicators and $F_{i}$ refers to the ith principal components.

The covariance matrix *F* can be expressed as follows:

(25) $$D\left(F\right)=\left[\begin{array}{cccc} \lambda_{1} & & & 0\\ & \lambda_{2} & & \\ & & \cdots & \\ 0 & & & \lambda_{p} \end{array}\right]$$

D(F) is an orthogonal symmetric matrix with non-negative eigenvalues $\lambda_{1}>\lambda_{2}>\ldots >\lambda_{p}>0$, where p≤m. If the eigenvector corresponding to each eigenvalue is denoted as $l_{1},l_{2}\ldots ,l_{p}$, then

(26) $$l_{j}={\left(v_{1j},v_{2j}\ldots ,\;v_{pj}\right)}^{T}$$

because $l_{j}$ is a unit vector with length 1.

(27) $$||l_{j}||=1$$

For any eigenvalue $\lambda_{j}$, the unit eigenvectors are ±$l_{j}$ and the direction of each eigenvector is random, which explains why the *RSEI* model has two opposite results [28]. Although the direction of eigenvectors has no effect on eigenvalues and their contribution rates, it can influence the results when using principal components to fit the indicators. Random eigenvector directions directly lead to wrong results, especially in batch operations. Therefore, it is necessary to select the correct direction of feature vectors before aggregating indicators. We judged the eigenvector direction according to previous results and the component values of eigenvectors corresponding to *NDVI*. The model of improved indicator aggregation method is expressed as follows:

(28) $$S_{CEQI}{}_{1}=\{\begin{array}{c} v_{11}x_{1}+v_{12}x_{2}+\ldots +v_{1m}x_{m},v_{ndvi}>0\\ (v_{11}x_{1}+v_{12}x_{2}+\ldots +v_{1m}x_{m})*\left(-1\right),v_{ndvi}<0 \end{array}$$

where $FS_{CEQI}{{}_{1}}_{1}$ represents the ecological environmental quality of pixel *i* in a single phase; $v_{11},\;v_{12}$, and $v_{1m}$ indicate component values of indicators corresponding to the eigenvectors of *PC*1; $v_{ndvi}$ indicates the eigenvectors of *NDVI* of *PC*1; $\mathrm{and}\;x_{1},\;x_{2}$, …$x_{m}$ are ecological indicators.

##### Multi-Temporal Mean Method

Ecological environment quality index from a single image could only reflect the ecological environmental state at that moment. Using the mean values of all images of a year/season can reflect the eco-environmental state of the year/season more objectively and stably. Thus, we used the multi-temporal mean method to take the average value of the ecological environment index of the year/season/month as the annual/seasonal/monthly ecological environment quality index in this study.

The calculation model is as follows:

(29) $$S_{CEQI}{}_{i}=\frac{F_{11i}+F_{12i}+\cdots +F_{1ni}}{n}$$

where $S_{CEQI}{}_{i}$ represents the comprehensive ecological environmental quality index at pixel *i*, $F_{1ni}$ refers to the ecological environmental quality index at pixel *i* and time *n*, and *n* is the number of phases that participate in the calculation at pixel *i*.

## 3. Results

### 3.1. Results of EI Model

**Figure 4:**
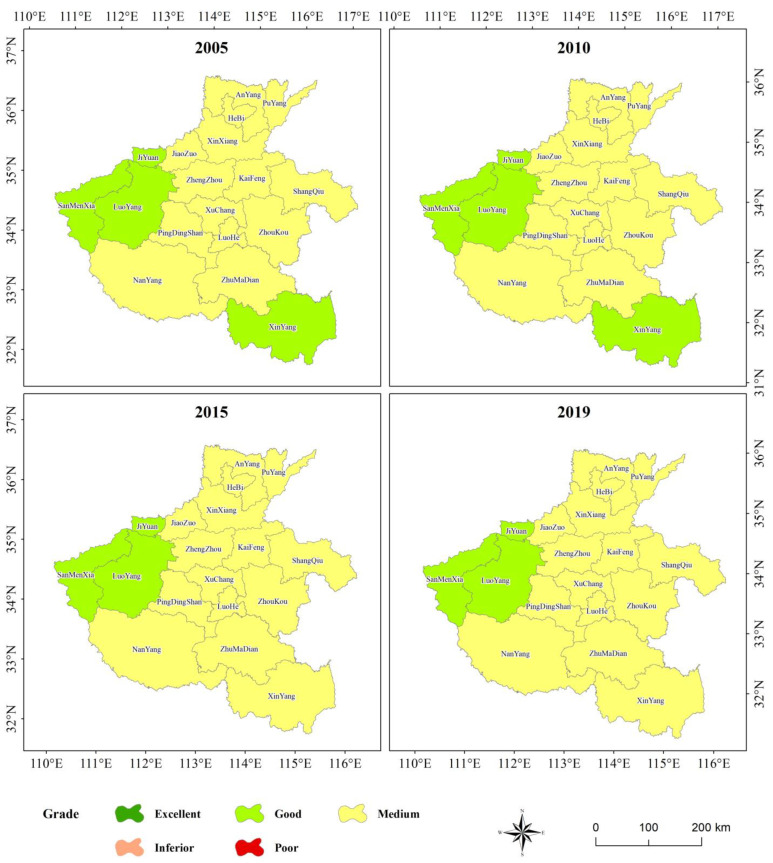
Eco-environmental quality level of the Henan Province using *EI* model.

The *EI* model was issued by the Ministry of Ecology and Environmental Protection of China aimed at evaluating the ecological status of administrative regions. It uses land use/cover secondary classification data, statistical data of administrative divisions, and remote images to obtain biological abundance, vegetation cover, water network density, land stress, pollution load, and environmental restriction indicators. These indicators are weighed and summed according to the corresponding weights in Regulations. Due to the lack of pollutant data in 1995 and 2000, in this study, we only calculated the *EI* index from 2005–2019. The results of the *EI* model are divided into different levels according to the grade range in Regulations. The results are shown in Figure 4.

The *EI* results showed that the ecological environmental quality of the Henan Province was mediocre and there were obvious geographical differences in the spatial distribution. The ecological environment of mountainous areas in the west was obviously better than that of plains in the Middle East. Zones with better ecological environmental quality were concentrated in TaiHang, FuNiu, TongBai, and DaBie Mountains. In terms of administrative regions, the ecological environment of SanMenXia, LuoyYang, and JiYuan Cities was generally better than that of other regions. The ecological environment of XinYang City deteriorated in 2015 and 2019, and the ecological level changed from good to medium.

Overall, *EI* could reflect the overall ecological environment of different administrative units and their changes. However, it requires statistical data on atmosphere, water, and air particulate matter, which are difficult to obtain. The spatial statistical unit of the model is usually an administrative division of a city/county, and the statistical time unit is year, which not only limits the time scale but also the space scale of research.

### 3.2. Results of EQI Model

**Figure 5:**
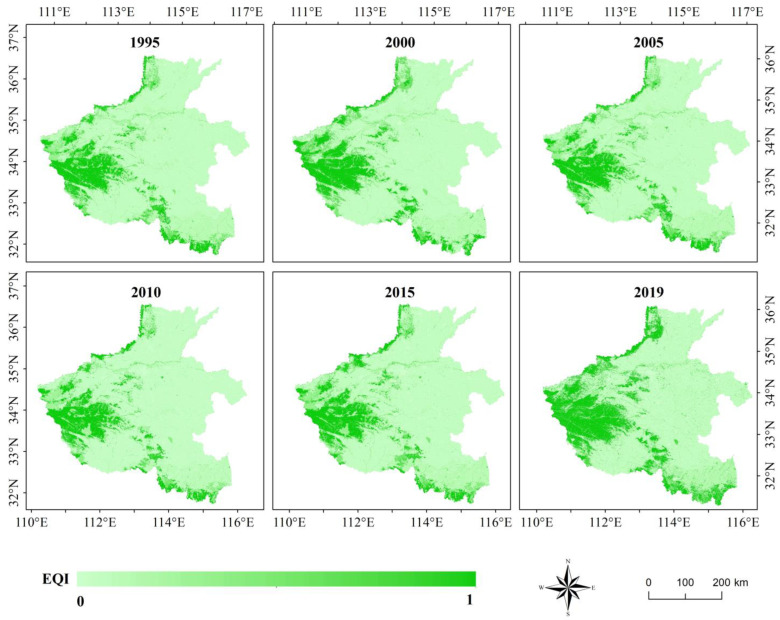
Ecological environmental quality index of Henan Province using *EQI* model.

The *EQI* model takes land use patches as the study unit and assigns the weight of secondary land use/cover type using the expert evaluation method to establish quantitative relationship between the land use type and ecological environment. We calculated the *EQI* of Henan Province in six periods according to previous studies [24]. The results are shown in Figure 5.

The results of *EQI* model revealed noticeable geographical differences in the spatial distribution of ecological environmental quality in the Henan Province. The ecological environment of the TaiHang Mountains in the north, TongBai and DaBie Mountains in the south, and FuNiu Mountains in the west of the study area were noticeably better than that of the Huang-Huai-Hai Plain in the middle of the study area and NanYang Basin. Areas with high *EQI* values were concentrated in forest ecosystems and those with low *EQI* values were concentrated in the construction land and bare land ecosystems.

In conclusion, the *EQI* model used land use/land cover data to evaluate the ecological environmental quality, which breaks the restrictions of administrative divisions and ensures geographical continuity of the study area to a certain extent. It can also reflect the differences in ecological environment of different ecosystems objectively within the study area. However, the ecological weight of ecosystems in the model is set subjectively and it is difficult to express spatial heterogeneity of the same ecosystem due to the fixed weight.

### 3.3. Results of RSEI Model

**Figure 6:**
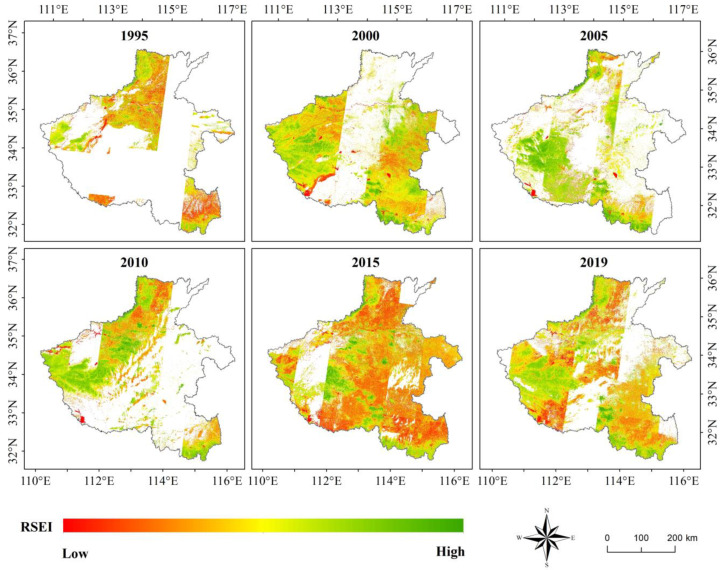
Ecological environmental quality index of the Henan Province using *RSEI* model.

*RSEI* is a model that uses four indices, *NDVI*, *NDBSI*, *Wet*, and *Lst*, calculated using a single remote sensing image in October to measure the annual regional annual ecological environment status. Due to the availability of data sources and objectivity of models, *RSEI* has been the most popular and widely used quantitative ecological and environmental quality model, and the *RSEI* results of Henan are shown in Figure 6.

Due to the influence of clouds, the images in October could not cover the entire study area; thus, there were large blanks in the calculation results and the calculation results of adjacent images had obvious boundaries. It proved that *RSEI* model was not suitable for a large study area. From the existing *RSEI* results, we could still roughly find that the ecological environmental status of western mountainous areas and border areas of the north and south of the Henan Province was better than that of central plains. However, the results showed no obvious difference between the cultivated land ecosystem and construction land ecosystem. This was because the main crops cultivated in Henan Province were wheat and corn in winter and summer, respectively. Winter wheat was harvested in October, while at the time, summer corn was still a seedling. The ecological environmental quality of cultivated land was the lowest in October throughout the year. Thus, it was unreasonable for the *RSEI* model to use a single scene image to measure the annual ecological environmental quality ignoring the phenological characteristics of plants.

### 3.4. Results of S_CEQI_ Model

**Figure 7:**
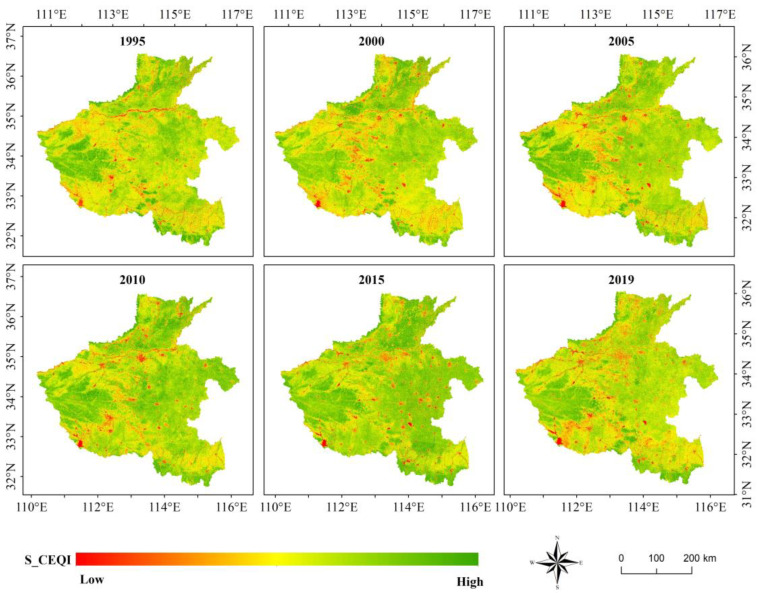
Ecological environmental quality index of the Henan Province using *S_CEQI_* model.

This study improved the data sources, dimensionless method, and index aggregation methods and introduced the multi-temporal average method to build *S_CEQI_*. It could overcome the limitation of area size and considered the phenological characteristics of plants. It is more objective and accurate to use the annual/seasonal/monthly average method to evaluate the regional annual/seasonal/monthly ecological environmental state. Moreover, due to improvement of the dimensionless method, the long-term sequence ecological environment evaluation was more objective and accurate. The *S_CEQI_* model calculated the annual/seasonal/monthly ecological environment quality index based on all good quality images and pixels throughout the year/season/month and then took the average of each pixel value as the final comprehensive ecological environmental quality index. The results are shown in Figure 7.

The *S_CEQI_* results showed obvious geographical differences in the spatial distribution of ecological environmental status in the Henan Province. The high value zones of eco-environment quality index were distributed in mountainous and hilly areas such as the TaiHang Mountains in the north, TongBai and DaBie Mountains in the south, and Funiu Mountains in the west of the study area. The ecological environmental quality of plain area was deteriorated compared to that of mountainous and hilly areas, and the ecological environmental quality of urban construction land was the worst. However, the results of *EI* and *EQI* were different from those of *S_CEQI_*, and they did not exhibit obvious low and high value clusters in the Henan Province. This is because *S_CEQI_* considered all phenological periods of vegetation comprehensively and expressed ecological differences in different regions in the form of mean values. There was a large amount of cultivated land in the middle and eastern parts of the Henan Province where the main crop was winter wheat. The ecological value of winter wheat in winter was much higher than that of deciduous broad-leaved forests of mountains to the west and along the northern and southern boundaries of study area. The multi-temporal averaging method averages the values of different seasons and neutralizes the ecological gap between two land types according to the actual situation of different periods.

### 3.5. Analysis of Results

#### 3.5.1. Consistency Analysis

According to the spatial distribution characteristics of ecological environmental quality calculated using the four models, *EI* (Figure 4), *EQI* (Figure 5), *RSEI* (Figure 6), and *S_CEQI_* (Figure 7) had the same spatial characteristics. They all proved that the ecological status was better in the west and northern and southern edge mountains and worse in the east and center and eastern plain areas.

Due to the absence of *RSEI* results in large areas, we ignored *RSEI* model in the overall comparison. Although the coupling variables of *S_CEQI_* and *EI* were different, the calculation results of *S_CEQI_* were numerically closer to those of *EI* (Table 5). Compared to *EI* results, the absolute maximum and minimum deviations of *S_CEQI_* and *EI* were 0.021 and 0.006, respectively, and the maximum and minimum deviations of *EQI* and *EI* were 0.124 and 0.062, respectively. The average offset of *EQI* was 7.236 times that of *S_CEQI_*. According to the profile in Section 3.5.2, the results calculated using the *EQI* model were significantly higher than those of *EI* and *S_CEQI_* in the deciduous broad-leaved forest ecosystem, mixed coniferous and broad-leaved forest ecosystem, and evergreen coniferous forest ecosystem (mainly bamboo forest) but lower than those of *EI* and *S_CEQI_* in the cultivated land ecosystem. This indicated that the *EQI* model overestimated the environmental quality of woodland ecosystems and underestimated the environmental quality of cultivated land ecosystems.

#### 3.5.2. Spatial Accuracy Analysis

##### Spatial Resolution Analysis

**Figure 8:**
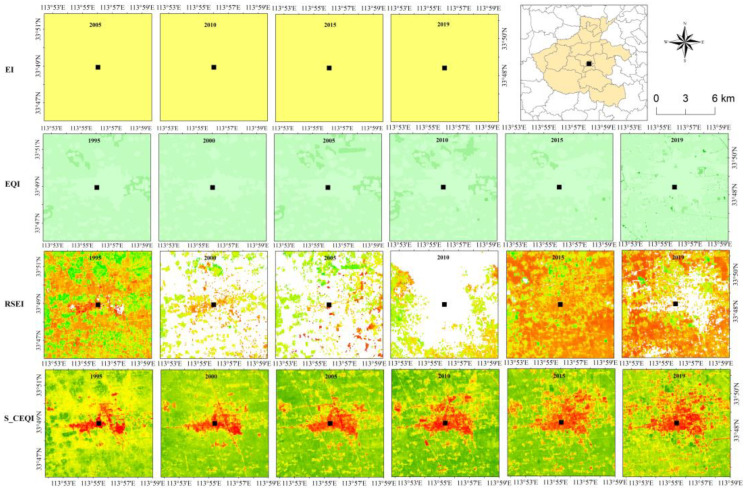
Spatial resolution of models (*EI*, *EQI*, *RSEI*, *S_CEQI_*).

A 10 km × 10 km sample was selected to compare the spatial resolution of the four models. The result (Figure 8) shows that the spatial resolution of the four models showed *S_CEQI_* = *RSEI* > *EQI* > *EI*. The *EI* result of the sample was a constant value without any fluctuation. The *EQI* result was either 0.2 or 0.4 with no continuous fluctuation that violates the geospatial continuity. Although the spatial resolution of *RSEI* was as high as *S_CEQI_* and its result was continuous spatially, its result appeared large gaps caused by the single image. It is clear that the *S_CEQI_* model performs best, and the results are complete, continuous and fine spatially.

##### Profile Analysis

**Figure 9:**
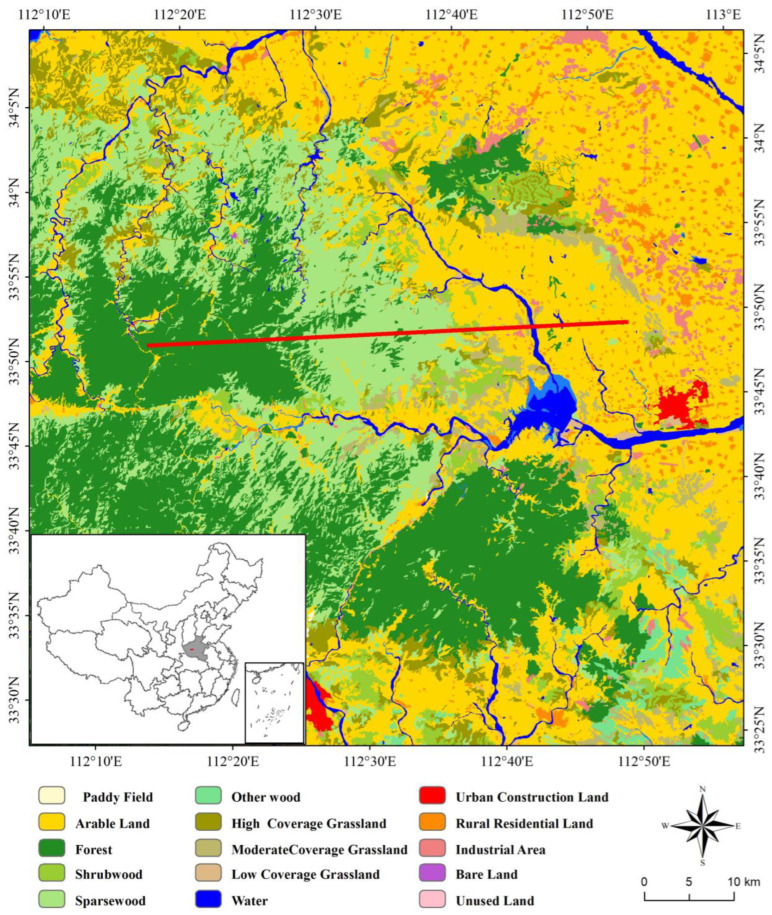
Profile position of the study area.

**Figure 10:**
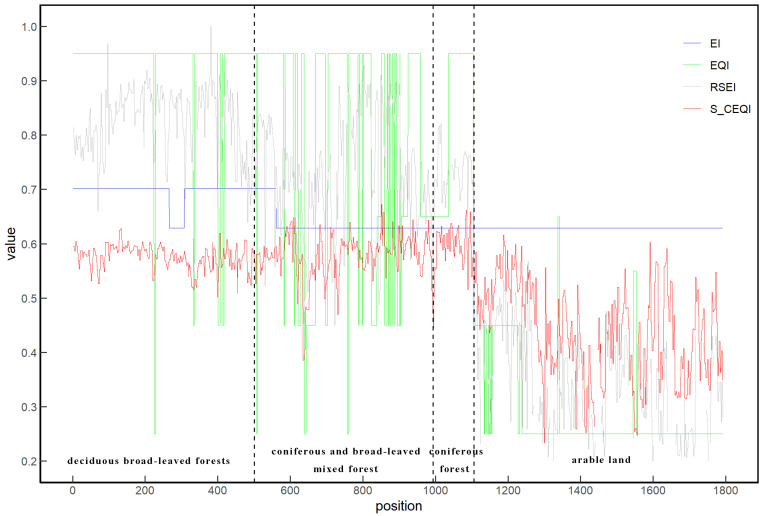
Profile map of the results of the three models (*EI*, *EQI*, *RSEI*, and *S_CEQI_*).

To compare differences in the results of different ecosystems under different models, this study selected the profile line (Figure 9) of transition zone from the forest to cultivated land for analysis. From the west to east, the types of ecosystems were deciduous broad-leaved forest, mixed coniferous and broad-leaved forest, evergreen coniferous forest, and cultivated land. The results of the profile using different models are shown in Figure 10.

The results of all four models proved that the ecological status in the west (forest ecosystem) was better than those in the east (arable land ecosystem); however, there were significant differences in the results of different models. Due to different data sources and mechanisms of the four models, the *EQI* results were quite different in the local spatial distribution. The *EI* model considers the administrative division as the research unit and spatial accuracy as size of the administrative unit. The *EQI* model considers the land use patch as the study unit and the spatial accuracy was equal to the size of land use patch. *RSEI* and *S_CEQI_* used the remote sensing image pixel as the calculation unit; thus, spatial resolution was the size of the remote sensing image pixel, which was 30 m in the study.

The measurement results of *EI* and *EQI* revealed extreme fluctuations in space due to limitation of the rough study unit. However, the profile curves of *RSEI* and *S_CEQI_* were continuous and smooth in space, attributed to the fine research unit. The ecological environment is a continuous, gradual, and dynamic process, and it should not have sudden changes such as “cliff” in space. Obviously, the ecological environmental quality measured using *RSEI* and *S_CEQI_* was more refined, and it could reflect the spatial heterogeneity of ecological environment of the same ecosystem. For example, the density of deciduous broad-leaved forests, leaf area index, tree species, and different crops had different ecological values. However, the results of *RSEI* and *S_CEQI_* were also quite different. The *RSEI* values of forest ecosystem were significantly higher than those of *S_CEQI_* while the *RSEI* values of arable land were significantly lower than those of *S_CEQI_*. This is because *S_CEQI_* replaces the value of a certain phase in October in *RSEI* with the average of all phases of the year. The *EQI* of a single image can only represent the ecological environment state at the time of taking the image, and it is unreliable to represent the annual ecological environment state. Thus, in this study, *RSEI* significantly overestimated the ecological value of forest ecosystems and underestimated the ecological value of cultivated land ecosystems. The results of *S_CEQI_* apears more objective and credible.

##### Sample Analysis

**Figure 11:**
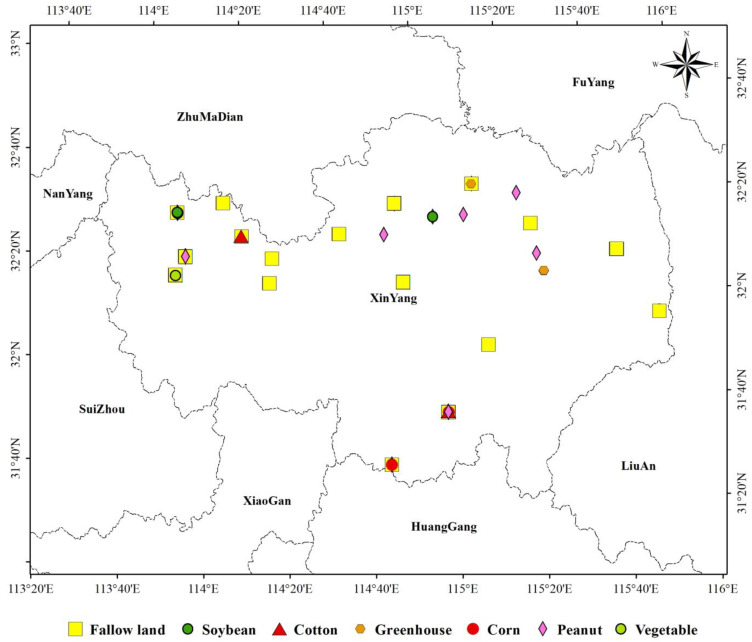
Spatial distribution map of cultivated land samples (Some samples are so close together that they cannot be distinguished on the map).

Considering cultivated land ecosystem as an example, this study selected a total of 219 points of seven crops, namely corn, soybean, cotton, greenhouse, peanut, vegetable, and fallow, to study the interpretation degree of differences in the ecological environment of the same ecosystem using the four models. The sample points consisted of 8 soybeans, 73 peanuts, 5 cotton, 36 vegetables, 9 greenhouses, 57 fallow lands, and 31 corns (Figure 11). All samples were distributed in Xinyang City, Henan Province, and all crops except greenhouses (winter fallow) were single-season crops.

Using the four models, we calculated *EQI*s of the 219 points as well as the Max, Min, Mean, Standard Deviation, and Variance. Statistical results (Table 6) revealed that the *EI* values of all 219 plots were 0.59 and *EQI* values were 0.25, without any fluctuations. The values of *RSEI* and *S_CEQI_* were relatively discrete. Further, the maximum value, minimum value, mean value, and standard deviation of *RSEI* were 0.67, 0.19, 0.41, and 0.17, respectively, and those of *S_CEQI_* were 0.62, 0.28, 0.46, and 0.06, respectively. The standard deviation of *RSEI* was larger than that of *S_CEQI_*, indicating that the ecological environmental index of crops in October fluctuated more than the annual average.

These differences reflect the different operating mechanisms of the models. The *EI* model used the administrative unit as the measurement unit, and the 219 plots were all located in Xinyang City, Henan Province (Figure 11). Therefore, the *EI* value of the same administrative region is a fixed value that cannot distinguish the differences in the ecological environmental status of samples (different crops of the same ecosystem). The *EQI* model considers secondary land use patch as the research unit, and the 219 samples were all dry lands of the same ecosystem. According to the expert scoring result, the ecological environmental quality of dry land was 0.25; thus, *EQI* values of the 219 samples were constant (0.25), which could not distinguish the ecological differences of different crops. However, *RSEI* and *S_CEQI_* take the pixel of image as the research unit whose spatial scale was fine enough to break the spatial restrictions of administrative divisions and land use patches.

**Figure 12:**
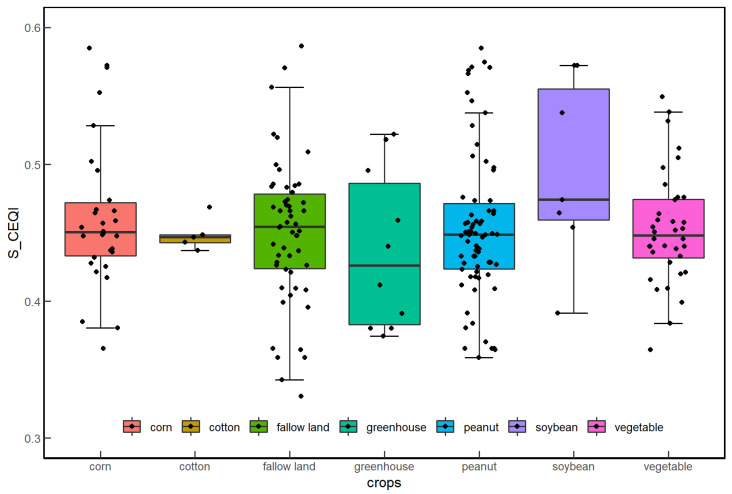
Box plots of integrated *EQI* using *S_CEQI_* of the 219 sample points.

As *RSEI* reflects the ecological environment at a certain moment, this study used the results of *S_CEQI_* model to study the differences in *EQI* of different crops. This study used boxplots (Figure 12) to explore the distribution characteristics and dispersion of *EQI* of different crop types. The average level and fluctuation degree of *EQI* of different crops were different. The median values of peanuts, cotton, vegetables, fallow lands, and corn were relatively close, but the median value of soybeans was significantly higher and the median value of greenhouses was significantly lower. The *EQI*s of different crops fluctuated at different degrees. The fluctuation degrees of soybean and greenhouse were noticeably larger than those of other crops. The *EQI*s of the same crops were also quite different due to influence of factors such as location, nature environment, and human activities, and *S_CEQI_* could accurately express such differences.

#### 3.5.3. Precision Analysis of RSEI and S_CEQI_

**Figure 13:**
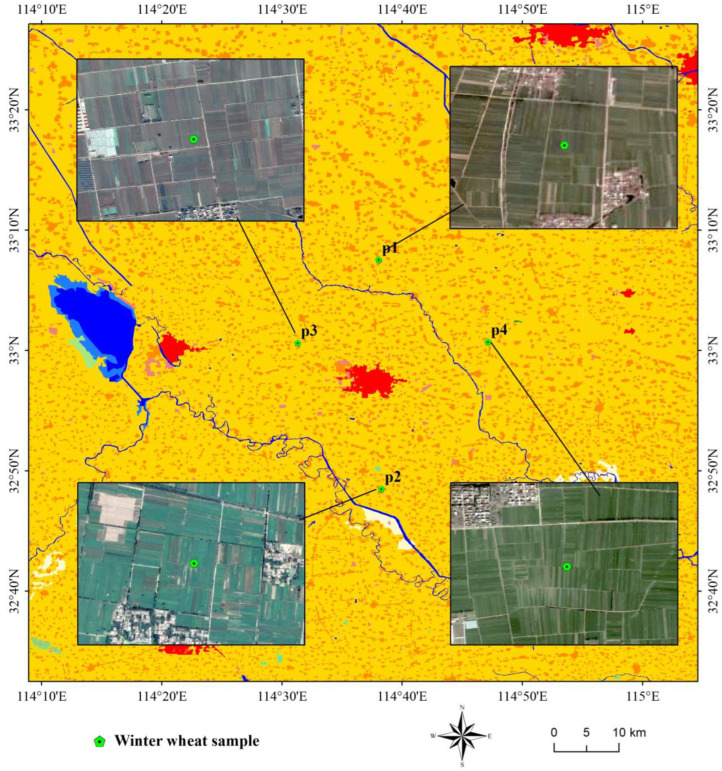
Spatial distribution of winter wheat samples.

Both *RSEI* and *S_CEQI_* exhibited high spatial accuracy and ensured continuity of geographical space, and they could express the ecological environmental quality of geographical space scientifically. Due to differences in data sources, *RSEI* adopted the static dimensionless method while *S_CEQI_* used the dynamic dimensionless method. We selected four winter wheat samples (Figure 13) and calculated the *EQI* of different phenological stages using *RSEI* and *S_CEQI_*, respectively, to compare the accuracy of the model. In this study, we compared four phenological stages of winter wheat, the jointing (23 August 2018), heading (8 April 2018), grain filling (10 May 2018), and maturity stages (11 June 2018).

**Figure 14:**
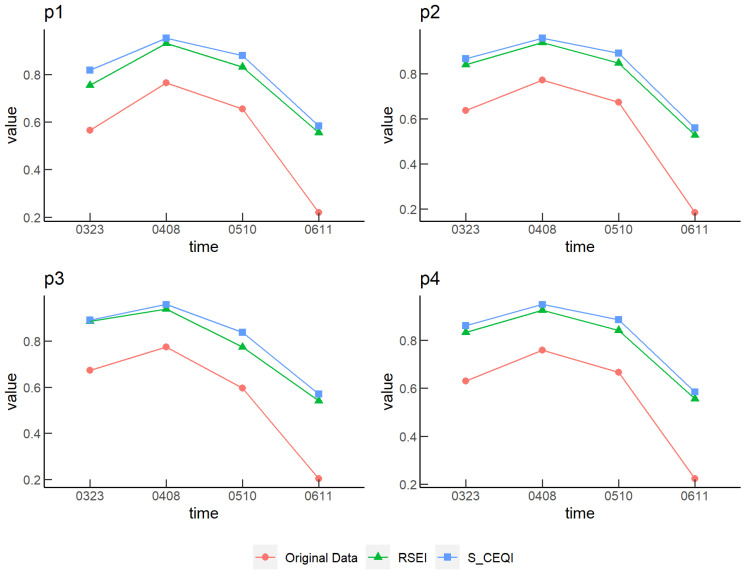
Comparison of different dimensionless results.

The results are shown in Figure 14. The original data indicated the data before dimensionalization, *RSEI* is the result of static dimensionless, and *S_CEQI_* is the result of full-sequence dimensionless. The time series trend of the result of static dimensionless and dynamic dimensionless of p1, p2, p3, and p4 was consistent with the original data, and the information on original data was well preserved. Thus, the static and dynamic dimensionless method could preserve incremental information of the same point at different times. However, as seen from Table 7, the original data of p1 on May 10 and p4 on March 23 were 0.656 and 0.629, respectively, and the corresponding static dimensionless results were 0.831 and 0.832; evidently, the relative magnitude between the two values changed after static dimensionless. The original data of p1 on May 10, which was greater than that of p4 on March 23, was smaller than that of p4 on March 23 after static dimensionless. However, *S_CEQI_* using full-sequence dimensionless method did not have such errors. It not only saved the incremental information of the same point at different times but also saved the incremental information between different points at different times.

#### 3.5.4. Temporal Resolution Analysis

**Figure 15:**
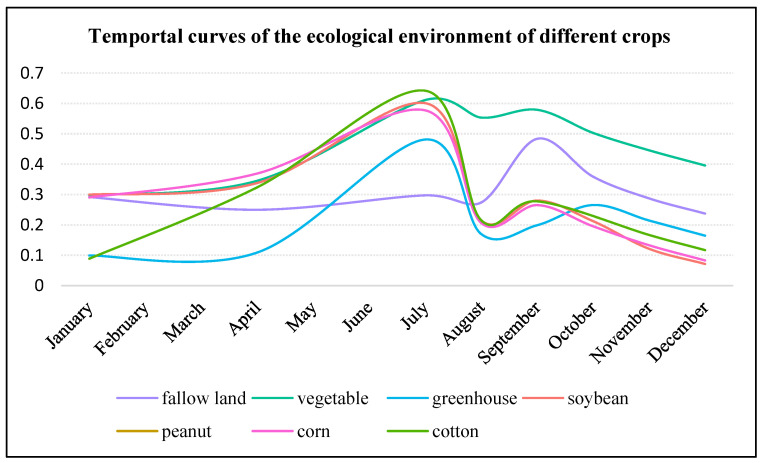
Time sequence diagram of ecological environmental quality change of different crop plots.

Temporal accuracy depends on the repetition period of data source of the model. Land use data was the main data source of *EI* and *EQI* models. However, currently, the land use data is usually updated annually or every 5 years. Therefore, the minimum time interval for recalculation of *EI* and *EQI* was 1 year. Thus, the *EI* and *EQI* models with annual recalculation could not estimate the ecological status and pixel changes in a year. Different from *EI* and *EQI*, remote sensing images with a short time cycle were the data source for *RSEI* and *S_CEQI_* models. In this study, the *RSEI* and *S_CEQI_* models used the Landsat satellite remote sensing images, whose revisit period was 16 days, to evaluate the regional ecological quality index; thus, the highest time accuracy of *S_CEQI_* and *RSEI* model was 16 days. *RSEI* is a static measurement model that uses a single image to evaluate the ecological environment status; thus, its results are static and cannot estimate changes in the ecological status. However, the *S_CEQI_* model used remote sensing big data and dynamic dimensionless method to accurately measure the continuous ecological environment changes. To verify the temporal characteristics of *S_CEQI_* model, the 219 samples were re-used to calculate the monthly average value of each crop to compare temporal differences of the eco-environment, and the results are shown in Figure 15.

The time series curves of *EQI*s of the seven crops were quite different due to different phenological characteristics. The time curves of ecological environmental quality index of corn, peanut, soybean, and cotton had similar phenological characteristics and changed trend. They were all planted in April and harvested in August. The ecological environmental quality indices of these crops reached the peak in July, proving that the ecological status was the best in July. The sharp decrease in August was attributed to human harvesting activities. The *EQI* of greenhouses reached two peaks in August and October, attributed to plantation of some off-season crops in winter (after October). The fallow land was dominated by grass that grows best in September; thus, its ecological environmental quality index reached the peak in September. Vegetables are a cash crop significantly affected by human activities. Its *EQI* was high due to economic reasons, and its curve also had double peaks. Thus, the ecological environment status of different crops was closely related to their phenological characteristics. The ecological environmental quality of different crops in the same period as well as the ecological environmental quality of the same crops in different periods were different.

In conclusion, the eco-environmental quality curves of different crops verified that the eco-environmental quality status of vegetation in different phenological stages was different, which proved that *S_CEQI_* model could evaluate regional eco-environmental status objectively by comprehensively considering each phenological characteristic of vegetation. The model also exhibited fine time accuracy.

#### 3.5.5. Compare Analysis of Globe Spatial Autocorrelation of Change of Ecological Environment Index

**Figure 16:**
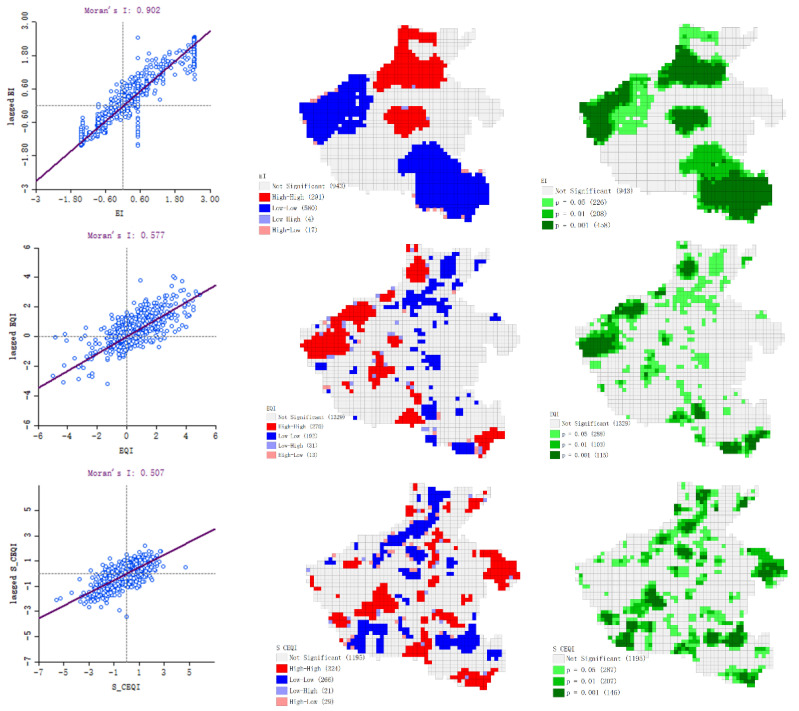
Spatial autocorrelation results showing Moran’s LISA cluster map, and LISA significance map of the change of ecological environment index from 1995 to 2019.

Moran scatter plots of the change of ecological environment index of Henan Province from 1995 to 2019 (*EI* was from 2005 to 2019) are shown in Figure 16. The high-high (HH), low-low (LL), low-ligh (LH), and high-low (HL) demonstrated significant spatial agglomerations. Because the result of *RSEI* was spatially incomplete, we only calculated the Moran’s Is of *EI*, *EQI* and *S_CEQI_*. The Moran’s Is were 0.902, 0.577 and 0.507, respectively, indicating the change of ecological environment index has obvious agglomeration in space. However, the spatial aggregation results of the three models are quite different. The Moran’s I was unusually large, and the significant aggregations were also too concentrated as a result from the roughness calculation unit of the *EI*. The cluster map of *EQI* showed that most of HH aggregations were distributed in the mountains and hilly areas were dominated by forest ecosystem, and most LL aggregations occurred in the plains where the main ecosystem was arable. Obviously, these aggregations relying on the ecosystem were not convincing. Different from *EI* and *EQI*, both HH and LL clusters of *S_CEQI_* were distributed in plains and Nanyang Basin where had strong human activities, what is consistent with objective fact that human activities have a significant impact on the ecological environment. This also proved that the *S_CEQI_* model was the most reliable.

## 4. Discussion

Ecological environment is the combined result of natural factors, social factors, economic factors, disasters, anthropogenic activities, and so on. It is a challenge to monitor the spatiotemporal changes of regional ecological status dynamically. In this study, we constructed a regional comprehensive eco-environmental quality index model (*S_CEQI_*) with spatiotemporal characteristics and compared its results with other models(*EI*, *EQI*, *RSEI*). The results showed that this model is superior to the other three models in terms of space, time and accuracy.

In the past, there was almost no long-term and continuous research of ecological environment evaluation, and most of their time intervals were 1 year, 5 years or longer. So, we attempted to carry out intensive time-interval ecological environment studies by replacing the data source with remote sensing big data who has the characteristics of long time series and short period. However, remote sensing big data also puts forward requirements for the stability, accuracy, and intelligence of the model. Because the remote sensing images of different rows and columns have different spatiotemporal information.

Therefore, establishing the association among different spatio-temporal images is the key to using remote sensing big data sources. In this study, the full sequence dimensionless method was used to achieve it. The full-sequence dimensionless method can effectively use each good quality pixel, which greatly reduces the influence of cloud. Besides, the spatial association enables the model to be applied in large areas which *RSEI* could not.

As a data source, remote sensing big data needs to be calculated in batches, which requires the model to be stable and intelligent. Therefore, we corrected the unstable factors of the index aggregation method in *RSEI*. The principal component analysis method is to assign weight to the ecological indicators according to their loads (contributions) in the first principal component (*PC*1). However, because each *PC*1 has two opposite eigen vectors, resulting in two opposite results. And these two opposite results happen randomly in the aggregation of indices. Our previous studies have shown that the direction of indicators having a positive effect on the ecological environment is always the same, the directions of indicators having a negative effect on the ecological environment are always the same, and the directions of positive indicators are always opposite to the negative ones in the eigenvectors of *PC*1 [28]. Based on the above-mentioned rules and the rule that *NDVI* always had a positive effect on the ecological environment, we fixed the weight of each indicator according to the direction of the eigen vector of *NDVI* in *PC*1. In fact, both *EI* and *EQI* models are subjective in setting the weight of ecological indices, and the weight of indicators has been controversial [37,38]. The automatic principal component index aggregation method is objective and repairs the instability of the model, making it possible to batch calculations with remote sensing big data.

Since remote sensing images are instantaneous, the calculation results of a single image are accidental. Using a single image to calculate the quality of ecological environment over a period is obviously not rigorous. The multi-temporal mean method was used in *S_CEQI_* to evaluate the ecological environment status and its changes in a certain period, for example, the annual, quarterly, and monthly, and the result showed to be objective and convincing.

All the four models could measure the regional ecological environmental quality and its changes to a certain extent. In this study, the measurement results of the four models were generally consistent. The *S_CEQI_* model was more precise than other models in spatial resolution, temporal resolution, and precision. However, there are still some uncertainties in the model, such as the impact of clouds. Although the full sequence dimensionless method can maximize the use of high-quality pixels in the image, the accuracy of the model results for those pixels that are frequently covered by clouds in the time series will also be affected. The multi-temporal mean method can reduce this uncertainty, but it cannot avoid it. Future research can interpolate or replace cloud data with other data sources to further improve the accuracy of the model. Besides, the ecological environment indices were still not rich enough. The ecological environmental quality status is the comprehensive result of direct and indirect interactions of many factors. The trial version of ‘Technical Specifications for Evaluation of Ecological Environment Status’ issued by the Ministry of Environmental Protection of China in 2006 and the updated published version in 2015 both considered the impact of air pollutants on the ecological environment. Moreover, the 2015 version added more air pollutants to the *EI* model, which proved that air quality is an important indicator of the ecological environment. Additionally, anthropogenic activities [39,40], topography [6], climate [41], population economy [41], and other factors have an increasingly significant impact on the ecological environment. Therefore, future studies should consider more complex ecological environment influencing factors to characterize the regional ecological environmental quality more objectively and accurately.

## 5. Conclusions

By analyzing the problems in the existing quantitative eco-environmental quality index model, we constructed an eco-environmental quality index model with spatiotemporal characteristics (*S_CEQI_*) by improving the data source, dimensionless method, index aggregation method of *RSEI*, and introduced the multi-temporal mean method to the model. We verified the reliability, spatial accuracy and temporal accuracy of *S_CEQI_* by comparing it with several popular quantitative models (*EI*, *EQI*, and *RSEI*). The main conclusions were as follows:

(1) The spatial distribution of the four models were consistent, indicating the *S_CEQI_* proposed in this study was reasonable.
(2) The resolution of *S_CEQI_* model was finest among the four models. Both profile analysis and sample analysis proved *S_CEQI_* could not only express the geographic spatial differences of ecological environment between different ecosystems but also that between the same ecosystems, which other models could not achieve.
(3) The *S_CEQI_* model broke the limitations of the study area and research time due to the improvement of the full-sequence dimensionless method who could establish an effective association of different spatio-temporal images.
(4) The *S_CEQI_* could carry out ecological environment research of long time series and dense time intervals due to the improvement of remote sensing big data. The curves of *S_CEQI_* of different crops demonstrated obvious differences among different phenological stages, indicating *S_CEQI_* has fine temporal resolution.
(5) Compare analysis of globe spatial autocorrelation of the change of ecological environment index showed that only the results of *S_CEQI_* were consistent with the fact that human activities have a significant impact on the ecological environment, which once again verified the precision of the model.

The *S_CEQI_* model could obtain regional ecological environment status timely, accurately, and dynamically, attributing to the improvement of data source and methods. It can not only provide objective and accurate data support for policy-making departments but also contribute to advancing the Sustainable Development Goals (SDGs). However, ecological environment is a complex system with many influencing factors. And this paper focused on the improvement of the method without the discussion of the influencing factors. Therefore, the indicator system and driving mechanism of ecological environment should be studied according to regional characteristics in future research.

## Figures and Tables

**Table 1:** Information on the datasets used in this study.

Data Set	Spatial Resolution	Temporal Resolution	Data Sources
Landsat 5	30 m × 30 m	16-day	https://glovis.usgs.gov/ (accessed on 17 Marth 2022)
Landsat 8	30 m × 30 m	16-day	https://glovis.usgs.gov/ (accessed on 17 March 2022)
LUCC	30 m × 30 m	5-year (1995, 2000, 2005, 2010, 2015, 2019)	Data Center of Middle&Lower Yellow River Regions, National Earth System Science Data Center, National Science&Technology Infrastructure of China (http://henu.geodata.cn, accessed on 17 March 2022)
NCEP/NCAR Reanalysis Data, Water Vapor	278.3 km×278.3 km, Bilinear resampling to 30 m× 30 m	6-h (data of 0:00 universal time when closest to landsat transit time was selected)	https://psl.noaa.gov/data/gridded/data.ncep.reanalysis.html/ ( accessed on 17 March 2022), doi:10.1175/1520-0477(1996)077<0437:TNYRP\>2.0.CO;2.
COD, NH_3_, SO_2_, YFC, NOX, SOL	Administrative city	1-year	Department of Ecological Capability of Henan Province

**Table 2:** Indicators and their weights of *EI*.

Indicator	Biological Abundance (*BA*)	Vegetation Cover (*NDVI*)	Water Density (*WD*)	Land Stress (*LS*)	Pollution Load (*PL*)	Environmental Limitation (*EL*)
Weight	0.35	0.25	0.15	0.15	0.10	Restrictive indicator

Note: The weight comes from Technical Specification for Assessment of Ecological and Environmental Conditions (HJ 192–2015).

**Table 3:** Ecological environmental status classification.

Level	Excellent	Good	Medium	Inferior	Poor
index	*EI* ≥ 75	55 ≤ *EI* < 75	35 ≤ *EI* < 55	20 ≤ *EI* < 35	*EI* < 20

**Table 4:** Land use classification system and its ecological environmental index.

First Land Use Type	Code	Secondary Land Use Type	Eco-Environment Quality Index	First Land Use Type	Code	Secondary Land Use Type	Eco-Environment Quality Index
Cultivated land	11	Paddy field	0.30	Construction land	51	Urban land	0.20
	12	Dry land	0.25		52	Rural settlement	0.20
					53	Other construction land	0.20
Forest	21	Woodland	0.95				
	22	Shrub forest	0.65	Unused land	61	Sand	0.01
	23	Sparse Woodland	0.45		62	Gobi	0.01
	24	Other woodland	0.40		63	Saline-alkali soil	0.05
					64	Marshland	0.65
Grassland	31	High coverage grassland	0.75		65	Bare land	0.05
	32	Medium coverage grassland	0.45		66	Bare rock gravel land	0.01
	33	Low coverage grassland	0.20				
Water	41	Rivers and Canals	0.55				
	42	Lake	0.75				
	43	Reservoir pond	0.55				
	44	Permanent glacial Snow	0.90				
	45	Tidal flat	0.45				
	46	Beach	0.55				

**Table 5:** Annual ecological environmental quality index of *EI*, *EQI*, and *S_CEQI_*.

Model	2005	2010	2015	2019
*EI*	0.479	0.486	0.489	0.473
*EQI*	0.366	0.364	0.365	0.411
*S_CEQI_*	0.500	0.500	0.496	0.491

**Table 6:** Descriptive statistics of the calculation results of *EQI*, *EI*, and *S_CEQI_* of 219 sample points.

Model	Max	Min	Mean	Standard Deviation	Variance
*EQI*	0.25	0.25	0.25	0.00	0.00
*EI*	0.59	0.59	0.59	0.00	0.00
*RSEI*	0.67	0.19	0.41	0.17	0.03
*S_CEQI_*	0.62	0.28	0.46	0.06	0.003

**Table 7:** Results of different dimensionless methods.

Point	Date	Original Data	*RSEI*	*S_CEQI_*
**p1**	20180323	0.565	0.754	0.818
	20180408	0.764	0.930	0.953
	20180510	0.656	0.831	0.879
	20180611	0.220	0.555	0.583
**p2**	20180323	0.636	0.840	0.866
	20180408	0.771	0.938	0.958
	20180510	0.673	0.847	0.891
	20180611	0.185	0.527	0.560
**p3**	20180323	0.673	0.886	0.891
	20180408	0.774	0.941	0.960
	20180510	0.596	0.774	0.839
	20180611	0.202	0.540	0.571
**p4**	20180323	0.629	0.832	0.861
	20180408	0.759	0.925	0.949
	20180510	0.666	0.841	0.886
	20180611	0.222	0.556	0.585

## Data Availability

Not applicable.
